# Genome-Wide Epigenetic Landscape of Lung Adenocarcinoma Links HOXB9 DNA Methylation to Intrinsic EGFR-TKI Resistance and Heterogeneous Responses

**DOI:** 10.1200/PO.20.00151

**Published:** 2021-02-19

**Authors:** Sheng-Fang Su, Chia-Hsin Liu, Chiou-Ling Cheng, Chao-Chi Ho, Tsung-Ying Yang, Kun-Chieh Chen, Kuo-Hsuan Hsu, Jeng-Sen Tseng, Huei-Wen Chen, Gee-Chen Chang, Sung-Liang Yu, Ker-Chau Li

**Affiliations:** ^1^ Institute of Statistical Sciences, Academia Sinica, Taipei, Taiwan; ^2^ Graduate Institute of Oncology, National Taiwan University, College of Medicine, Taipei, Taiwan; ^3^ YongLin Institute of Health, YongLin Scholar, National Taiwan University, Taipei, Taiwan; ^4^ Bioinformatics Program, Taiwan International Graduate Program, Academia Sinica, Taipei, Taiwan; ^5^ Institute of Biomedical Informatics, National Yang-Ming University, Taipei, Taiwan; ^6^ NTU Centers for Genomic and Precision Medicine, National Taiwan University, College of Medicine, Taipei, Taiwan; ^7^ Department of Internal Medicine, National Taiwan University Hospital and National Taiwan University, College of Medicine, Taipei, Taiwan; ^8^ Department of Internal Medicine, Division of Chest Medicine, Taichung Veterans General Hospital, Taichung, Taiwan; ^9^ School of Medicine, National Yang-Ming University, Taipei, Taiwan; ^10^ Department of Applied Chemistry, National Chi Nan University, Nantou, Taiwan; ^11^ Institute of Biomedical Sciences, National Chung Hsing University, Taichung, Taiwan; ^12^ Internal Medicine, Division of Critical Care and Respiratory Therapy, Taichung Veterans General Hospital, Taichung, Taiwan; ^13^ Graduate Institute of Toxicology, National Taiwan University, College of Medicine, Taipei, Taiwan; ^14^ Division of Pulmonary Medicine, Department of Internal Medicine, Chung Shan Medical University Hospital, Taichung, Taiwan; ^15^ Institute of Medicine, Chung Shan Medical University, Taichung, Taiwan; ^16^ School of Medicine, Chung Shan Medical University, Taichung, Taiwan; ^17^ Department of Clinical Laboratory Sciences and Medical Biotechnology, National Taiwan University, College of Medicine, Taipei, Taiwan; ^18^ Department of Laboratory Medicine, National Taiwan University Hospital, Taipei, Taiwan; ^19^ Department of Pathology and Graduate Institute of Pathology, National Taiwan University, College of Medicine, Taipei, Taiwan; ^20^ Institute of Medical Device and Imaging, National Taiwan University, College of Medicine, Taipei, Taiwan; ^21^ Graduate Institute of Clinical Medicine, National Taiwan University, College of Medicine, Taipei, Taiwan; ^22^ Department of Statistics, University of California, Los Angeles, Los Angeles, CA

## Abstract

**PURPOSE:**

Epidermal growth factor receptor (EGFR)-tyrosine kinase inhibitors (TKIs) show efficacy in treating patients with lung adenocarcinoma with *EGFR*-activating mutations. However, a significant subset of targeted patients fail to respond. Unlike acquired resistance (AR), intrinsic resistance (IR) remains poorly understood. We investigated whether epigenomic factors contribute to patient-to-patient heterogeneity in the EGFR-TKI response and aimed to characterize the IR subpopulation that obtains no benefit from EGFR-TKIs.

**PATIENTS AND METHODS:**

We conducted genome-wide DNA methylation profiling of 79 tumors sampled from patients with advanced lung adenocarcinoma before they received EGFR-TKI treatment and analyzed the patient responses. Pyrosequencing was performed in a validation cohort of 163 patients with *EGFR*-activating mutations.

**RESULTS:**

A DNA methylation landscape of 216 CpG sites with differential methylation was established to elucidate the association of DNA methylation with the characteristics and EGFR-TKI response status of the patients. Functional analysis of 37 transcription-repressive sites identified the enrichment of transcription factors, notably homeobox (*HOX*) genes. DNA methylation of *HOXB9* (cg13643585) in the enhancer region yielded 88% sensitivity for predicting drug response (odds ratio [OR], 6.64; 95% CI, 1.98 to 25.23; *P* = .0009). Pyrosequencing validated that *HOXB9* gained methylation in patients with a poor EGFR-TKI response (OR, 3.06; 95% CI, 1.13 to 8.19; *P* = .019).

**CONCLUSION:**

Our data suggest that homeobox DNA methylation could be a novel tumor cellular state that can aid the precise categorization of tumor heterogeneity in the study of IR to EGFR-TKIs. We identified, for the first time, an epigenomic factor that can potentially complement DNA mutation status in discriminating patients with lung adenocarcinoma who are less likely to benefit from EGFR-TKI treatment, thereby leading to improved patient management in precision medicine.

## INTRODUCTION

Adenocarcinoma is the most common subtype of non–small-cell lung cancer (NSCLC), which has the highest cancer mortality worldwide.^1^ High intratumor heterogeneity of lung adenocarcinoma has been documented, and several targetable oncogenic mutations^2-9^ have been characterized. Inhibition of epidermal growth factor receptor (EGFR) kinase activity by EGFR-tyrosine kinase inhibitors (TKIs), such as erlotinib, gefitinib, and afatinib, was effective in patients with NSCLC with *EGFR*-activating mutations. However, despite the remarkable clinical success, the treatment efficacy was still limited to 50%-80%.^10,11^ The sizable percentage of nonresponders (20%-30%) suggested intrinsic TKI resistance and substantial heterogeneity among tumors, even among *EGFR*-mutant tumors, highlighting the need for reliable predictive biomarkers.

**CONTEXT**

**Key Objective**
A sizable portion (20%-30%) of patients with epidermal growth factor receptor (*EGFR*)–mutant non–small-cell lung cancer have no good initial clinical response to EGFR-tyrosine kinase inhibitors (TKIs), and how to predict intrinsic drug resistance accurately is challenging. Global DNA methylation landscape of tumors from patients with lung adenocarcinoma before TKI treatment was analyzed to investigate the association with EGFR-TKI responses.
**Knowledge Generated**
A total of 216 TKI response–associated methylated sites were identified, and functional analysis revealed the enrichment of homeobox genes. In particular, increased methylation of *HOXB9* correlated with higher rate of intrinsic resistance (IR) to EGFR-TKI.
**Relevance**
DNA methylation provides a different dimension to complement the DNA mutation–based markers for understanding the mechanism of IR. Evaluation of the methylation level on *HOXB9* may be incorporated in the management of lung adenocarcinoma to aid the prediction of EGFR-TKI response.

The comprehensive molecular profiling of pretreatment lung adenocarcinoma to identify inherently TKI-resistant cases can aid the development of potential strategies to manage such cases. Recently, genomic profiling of advanced NSCLC with *EGFR* mutations at baseline has identified multiple genetic, phenotypic, and functional mechanisms that may contribute to intrinsic resistance (IR).^12^ Whole-exome sequencing on untreated *EGFR*-mutant NSCLC tumors^13^ and the detection of co-occurring genetic alternations, such as *MET*, *PIK3KA*, *CDK4*, *CDK6*, and *NF1*, in the cfDNA of advanced-stage patients before treatment with EGFR-TKI^14^ suggest many DNA-based biomarkers for IR prediction. On the other hand, tumor suppressor genes involved in the alternative mechanisms of IR may be inactivated by epigenetic mechanisms that result in phenotypic or functional changes.^15^ However, although studies have reported that epigenetic changes in tumor participate in the evolution of acquired drug resistance through regulating gene expression patterns,^16^ epigenomic data associated with IR to TKI are lacking. Modification of methylation on DNA is stable and abnormal methylation represents an early event for cancer diagnosis, making methylation aberrations equally suitable candidates for recurrence detection and prediction of patient survival.^17-21^ Therefore, we undertook a genome-wide approach to investigate DNA methylation patterns associated with IR to TKI.

DNA methylation that occurs at cytosines of CpG dinucleotides, especially within CpG islands in the promoter region, can lock genes in off status, resulting in a transcriptionally silent state.^22,23^ Although DNA methylation is an important mechanism for maintaining normal development and cellular homeostasis, aberrant DNA methylation–mediated silencing of tumor suppressor genes has been reported to be associated with cell survival and progression in cancer.^24^ DNA methylation profiling of tumors, including those of glioma, acute myeloid leukemia, and colorectal and lung cancers, has aided the identification of cancer subtypes correlated with clinical outcomes.^25-29^

In this study, we aimed to identify epigenetic markers for predicting drug efficacy in patients with lung adenocarcinoma. We conducted genome-wide DNA methylation profiling of tumors from patients before their EGFR-TKI therapy and established a DNA methylation landscape to elucidate the association of DNA methylation with the EGFR-TKI response status of patients via a pipeline of statistical analysis, gene ontology (GO), and bioinformatics analysis. Our study identified a DNA methylation marker for predicting drug response in lung adenocarcinoma and provides insight into the epigenetic regulation of IR to EGFR-TKIs.

## PATIENTS AND METHODS

Tumor samples were obtained from two cohorts of patients with lung adenocarcinoma before EGFR-TKI therapy. Detailed accounts of sample collection and protocols for DNA extraction, bisulfite conversion, DNA methylation analysis, pyrosequencing along with statistical analysis, bioinformatics analysis, and data availability are presented in the Data Supplement.

## RESULTS

### Clinicopathologic Features of the Patients

Table 1 lists the clinicopathologic features of the two cohorts. Patients in the discovery cohort were at the advanced stage (IIIB or IV), and most *EGFR* mutations were L858R point mutations and exon 19 deletions. No significant differences were found in the distribution of tumor stage, sex, or smoking behavior between *EGFR* mutant and *EGFR* wild type. The validation cohort consisted of 163 *EGFR*-mutant patients and the majority (85.28%) were at stage IV. The TKI response assessment—progressive disease (PD), stable disease (SD), partial response (PR), or complete response (CR)—was determined according to the RECIST guidelines.^30^ The PD group, defined at the first scan done at 8 weeks following the start of EGFR-TKI, is considered as patients with IR. We determined the disease control rate (DCR) by comparing the number of patients with SD, PR, or CR with those with PD to study intrinsic drug resistance.

**TABLE 1.**
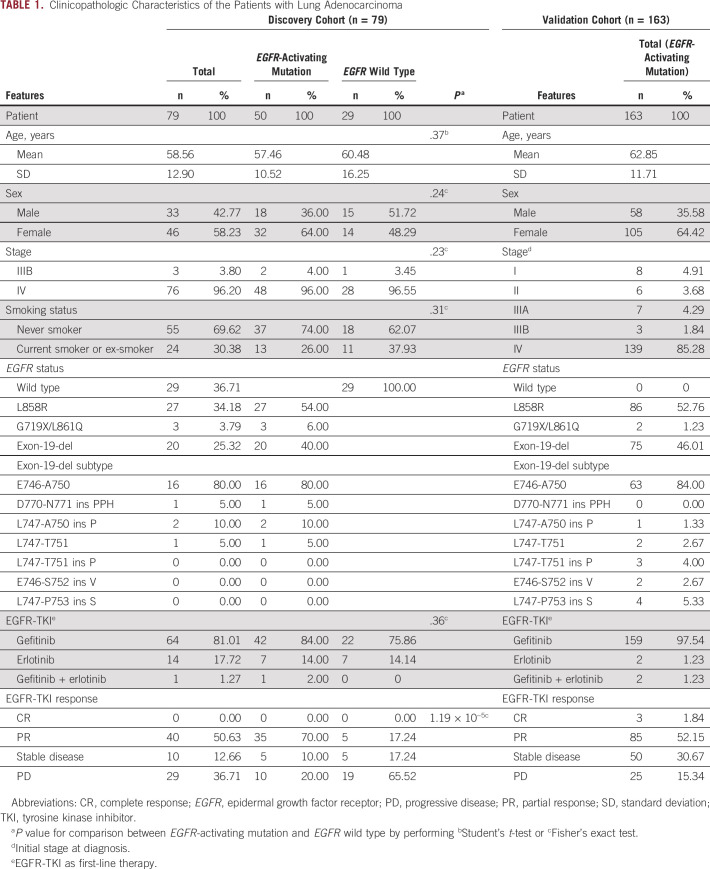
Clinicopathologic Characteristics of the Patients with Lung Adenocarcinoma

### Differential DNA Methylation Sites Associated With EGFR-TKI Response Heterogeneity Were Enriched in Transcription Factors

**FIG 1.**
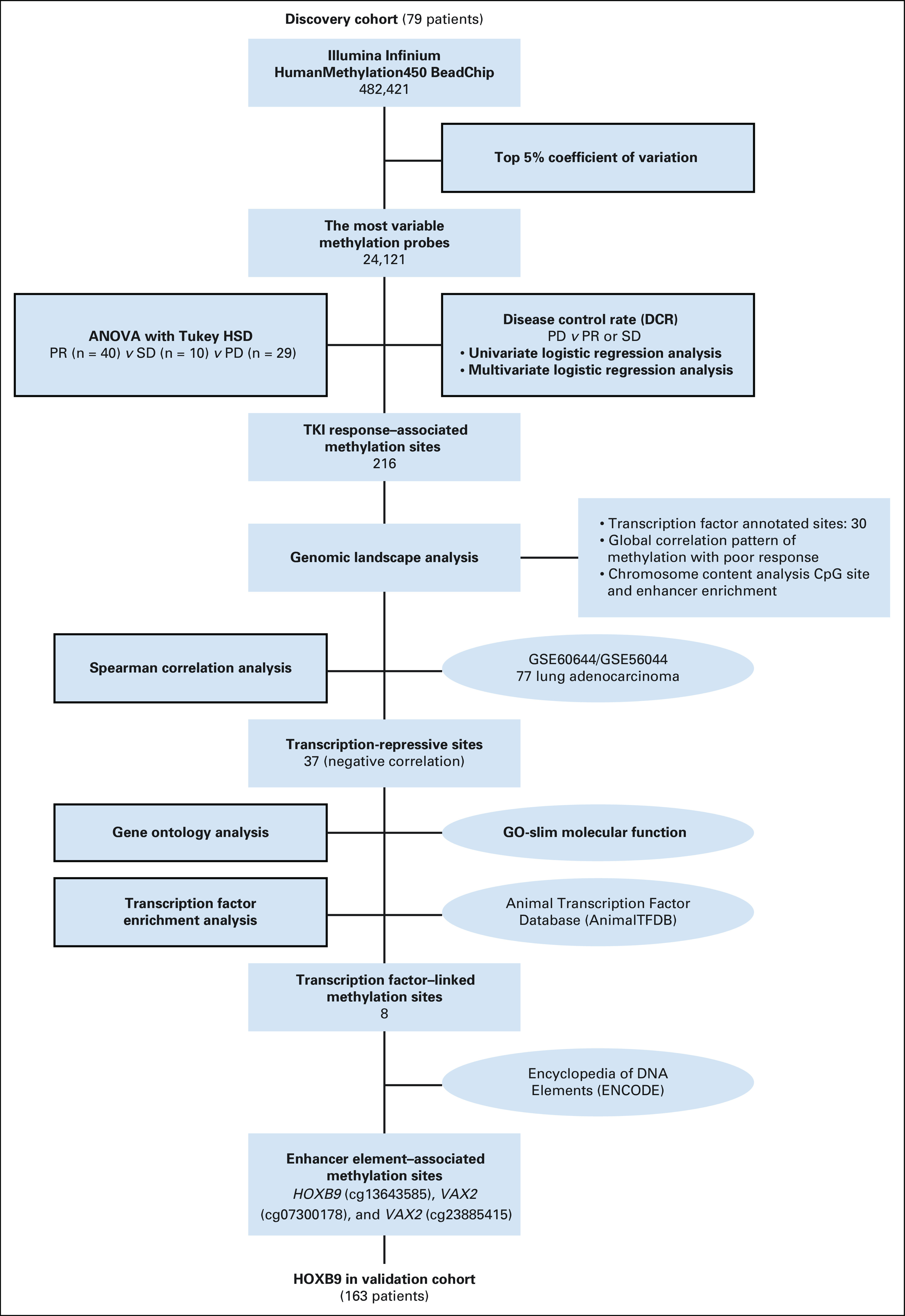
Flowchart of the study design and discovery cohort analysis. Whole-genome DNA methylation analysis was conducted in 79 patients to investigate the association of DNA methylation with EGFR-TKI response. Starting from a total of 482,421 CpG probes, a series of statistical and bioinformatic procedures were conducted to filter out the less-relevant probes. The numbers show the number of CpG probes remaining after passing each selection procedure. Statistical tests are shown in the blocks with black borders. Publicly available databases are shown in the ovals in the diagram. ANOVA, analysis of variance; DCR, disease control rate; EGFR, epidermal growth factor receptor; HSD, honestly significant difference; PD, progressive disease; PR, partial response; SD, stable disease; TKI, tyrosine kinase inhibitor.

Following the flowchart in Figure 1, the DNA methylation profiles of the 79 tumors in the discovery cohort were assessed. Only probes showing a high variation across tumors were retained. Using the top 5% coefficient of variation as the cutoff, 24,121 probes with the greatest variability were analyzed, and 391 probes were found correlated with EGFR-TKI response. To identify the subpopulation of intrinsic drug-resistant patients obtaining no benefit from TKIs, we further analyzed DCR by univariate and multivariate logistic regression. Of 391 probes, 216 were found significant (Data Supplement). Interestingly, 30 of the 216 probes (13.88%) were annotated to transcription factors (TFs). Comparison with the percentage of probes in the Illumina Infinium HumanMethylation450 BeadChip annotated to TFs (34,129/482,421; 7.07%) revealed that the enrichment was highly significant (binomial *P* = .0001).

### DNA Methylation Landscape of 216 Probes Associated With Differential EGFR-TKI Responses

**FIG 2.**
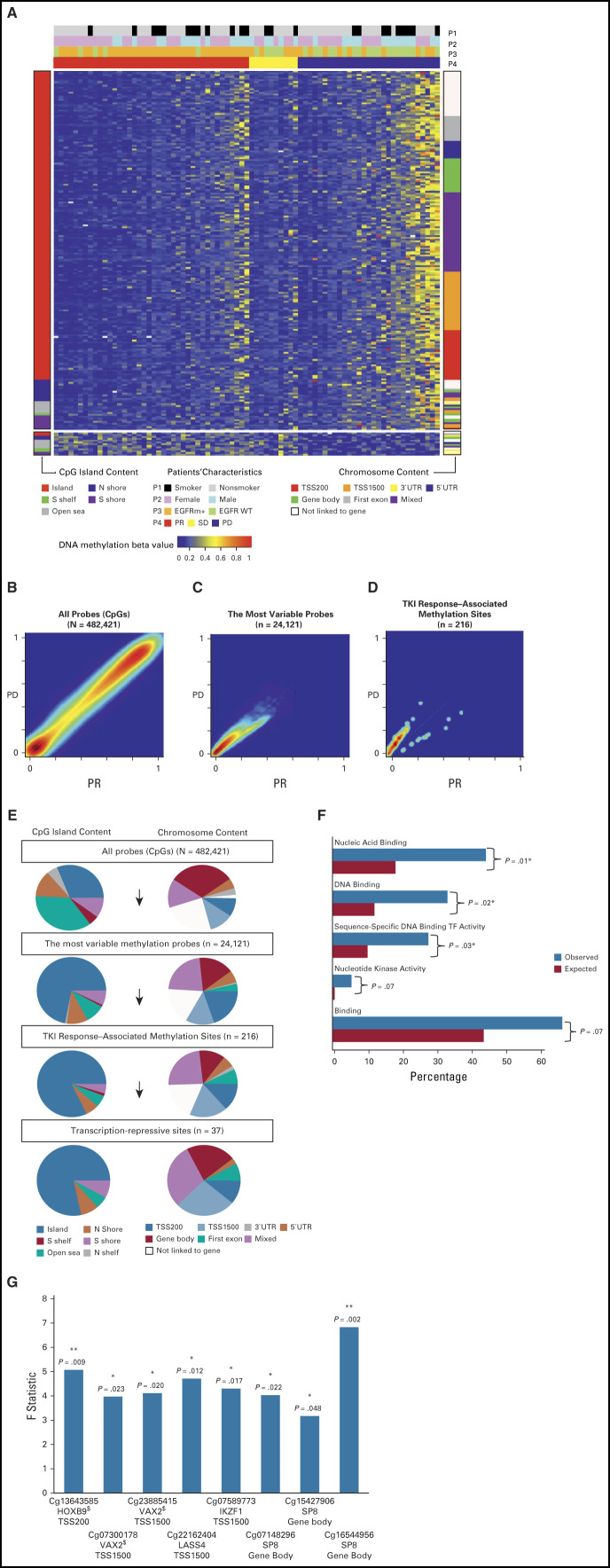
DNA methylation landscape of 79 patients with lung adenocarcinoma with different EGFR-TKI responses. (A) DNA methylation heatmap of 216 EGFR-TKI response–associated sites for DCR prediction. The color-coded beta value at each selected probe ranged between 0 (blue) and 1 (red). The patients’ characteristics, smoking behavior (P1), sex (P2), *EGFR* status (P3), and EGFR-TKI response (P4), are shown by the bars at the top of the heatmap. Probes were grouped by CpG island content (left bar) and by chromosome content (right bar). (B-D) DNA methylation density for PD versus PR. For each CpG probe, the average beta value across patients with PD was plotted against that across patients with PR and is shown as a smooth kernel scatter plot. The set of probes used is indicated at the top of each plot, along with the number of probes. In the gradient scale, red represents the densest region, whereas purple represents the sparsest region; PR (red), SD (yellow), and PD (blue). (E) Genomic context distributions of CpG methylation sites. The probe distributions in the CpG context and the gene context are shown on the left and right, respectively, for all probes in the array (top), for the most variably methylated probes (upper middle), for the TKI response–associated methylation sites (lower middle), and for the transcription-repressive sites (bottom). (F) Functional enrichment analysis of genes linked to the 37 transcription-repressive sites. All 186 molecular function categories in GO-slim were evaluated, and the top 5 most enriched categories are shown, along with fold changes and *P* values. The GO terms involving transcription factors were GO:0003676, GO:0003677, and GO:0003700. (G) F value of ANOVA for the eight TF-linked sites. *P < .05; **P < .01; ^**$**^enhancer (ENCODE). ANOVA, analysis of variance; DCR, disease control rate; EGFR, epidermal growth factor receptor; ENCODE, Encyclopedia of DNA Elements; GO, gene ontology; PD, progressive disease; PR, partial response; SD, stable disease; TF, transcription factor; TKI, tyrosine kinase inhibitor.

The patient DNA methylation profiles with the 216 TKI response–associated methylated probes were established (Fig 2A). The majority of the probes (203) had higher DNA methylation in patients with a poor response than in those with a favorable response; hypermethylation correlated with poor response. Only a group of 13 probes (bottom of the plot) showed the opposite trend; hypomethylation correlated with poor response. The probes were grouped by their locations relative to the CpG island content and by the chromosome content relative to the transcription start site. We also rearranged this plot according to patient characteristics (Data Supplement) and conducted a correlation analysis by linear regression to find the significantly correlated probes (*P* < .05). We found 11 probes correlated with *EGFR* status, 54 probes with sex, and 32 probes with smoking behavior. A global view of the DNA methylation distribution contrasting the patients with PD against those with PR showed that all but 13 probes were located above the diagonal line, elucidating a clear pattern of methylation gain in patients with PD (Fig 2B-D). The tumors of patients who were more likely to be resistant to EGFR-TKIs tended to have higher pretreatment methylation levels.

### Chromosomal Context Analysis of Candidate CpG Sites Showed Enrichment in CpG Islands and Gene Promoter Regions

The CpG sites were assigned to the annotated categories according to their chromosome positions relative to the nearby transcription start sites and the closest CpG islands (Data Supplement). We examined the changes in the proportion of each category during our probe selection and found an increasing trend in CpG islands and the gene promoter region TSS1500 (between 1,500 bp and 200 bp upstream of the transcription start site). The 216 EGFR-TKI response prediction sites were highly enriched in CpG islands (81.94%) compared with only 31.15% sites initially in CpG islands. Similarly, the percentage of sites in the TSS1500 region increased from 11.65% to 18.06%. In addition, the proportion of probes in the open sea region decreased sharply from 35.89% to 5.56%. For the 37 transcription-repressive sites, the enrichment pattern in CpG islands was retained and that in TSS1500 was increased to 27.03% (Fig 2E).

### Identification of Transcription-Repressive Methylation Sites

We investigated the potential of the 216 methylation sites in cis-regulation of gene expression by correlating publicly accessible mRNA gene expression data (GSE60644) with DNA methylation data (GSE56044) in lung adenocarcinoma (Data Supplement). We computed the Spearman rank correlation between DNA methylation and gene expression to select the methylation sites that showed evidence of repressing downstream transcript expression. A total of 37 sites were identified as transcription-repressive sites (Data Supplement).

### Functional Enrichment Analysis of Transcript-Linked Methylation Showed Eight Probes Linked to TFs

To evaluate the molecular function of the genes mapped by the 37 transcription-repressive sites, GO enrichment analysis was conducted. Three top significant molecular function categories of GO slim terms, nucleic acid binding (GO:0003676), DNA binding (GO:0003677), and sequence-specific DNA binding TF activity (GO:0003700), were all related to TFs (Fig 2F and Data Supplement). Comparisons of the percentage of TFs in the 37 transcription-repressive sites (21.62%; 8/37) with that in all probes (7.07%; 34,129/482,421) and in the most variable probes (11.59%; 2,796/24,121) revealed significant enrichment (*P* values = .0025 and .036, respectively; Data Supplement). Furthermore, using Animal Transcription Factor Database (AnimalTFDB), we found that eight of the 37 sites were annotated to five TFs: IKZF1, HOXB9, SP8, LASS4, and VAX2 (Table 2 and Data Supplement). Figure 2G shows the F statistic and the corresponding *P* value from analysis of variance for each of the eight sites, along with the location and chromosome context information. We found that seven of the eight TF-linked sites are located in the context of CpG islands and five in the transcription start site (TSS) region (Table 2). Sites located in the TSS region were what we focused on next.

**TABLE 2.**
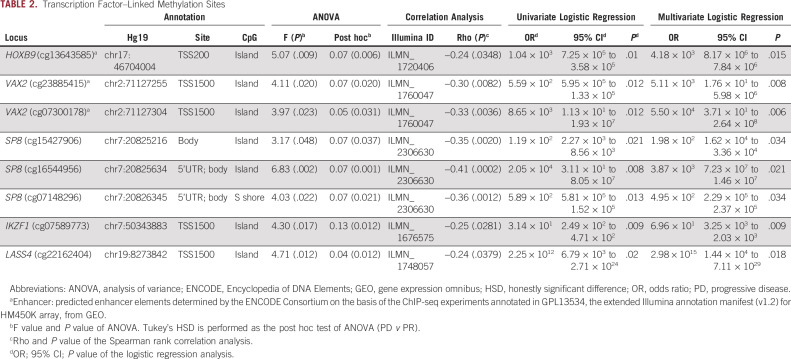
Transcription Factor–Linked Methylation Sites

### Identification of the DNA Methylation Site cg13643585 (*HOXB9*) With Predictive Ability Regarding EGFR-TKI Response

**FIG 3.**
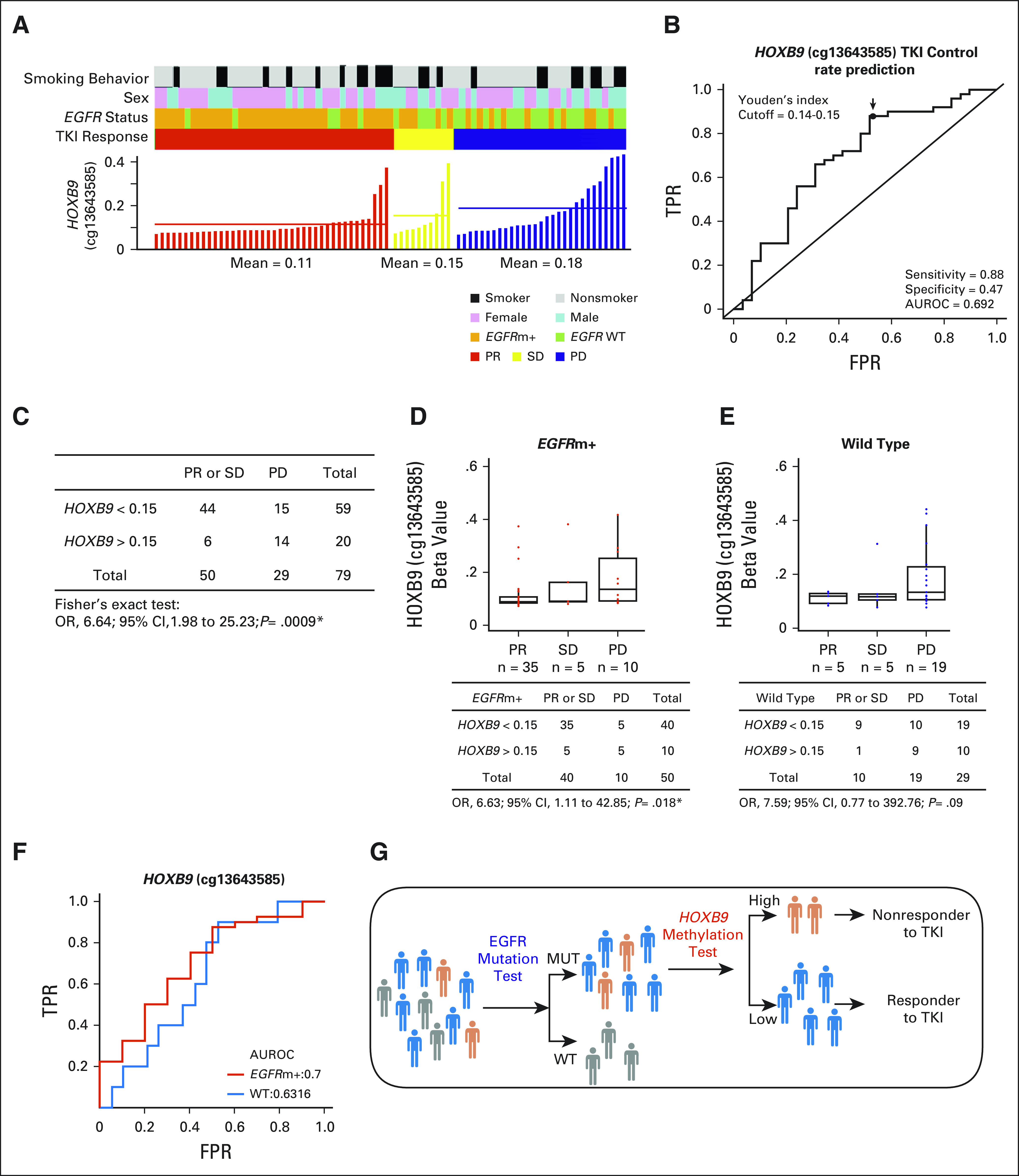
DNA methylation of *HOXB9* (cg13643585) was correlated with EGFR-TKI response. (A) The *HOXB9* beta values of 79 patients with NSCLC with PR (red), SD (yellow), and PD (blue). (B) AUROC and (C) Fisher’s test of *HOXB9* methylation for predicting the EGFR-TKI response. . The optimal cutoff points were determined by the Youden's index, which maximizes the sum of the specificity and sensitivity. (D-F) Stratified analysis. Patients were classified as *EGFR*m+ (red) or *EGFR* wild type (blue). Comparison of *HOXB9* beta value among PR, SD, and PD. (G) Strategy using *HOXB9* methylation complementing EGFR to classify the subpopulation of patients likely to be nonresponders to EGFR-TKI. AUROC, area under the receiver operating characteristic curve; EGFR, epidermal growth factor receptor; FPR, false positive rate; NSCLC, non–small-cell lung cancer; OR, odds ratio; PD, progressive disease; PR, partial response; SD, stable disease; TKI, tyrosine kinase inhibitor; TPR, true positive rate; WT, wild type.

Among the five sites in the TSS region, three sites, cg13643585 (*HOXB9*), cg07300178 (*VAX2*), and cg23885415 (*VAX2*), are located in enhancer regions annotated by the Encyclopedia of DNA Elements (ENCODE) Consortium. The DNA methylation levels of cg13643585, cg07300178, and cg23885415 were the highest in the group of patients with PD (Fig 3A and Data Supplement). *HOXB9* methylation (cutoff beta value = .15) predicted disease control by EGFR-TKIs with 88% sensitivity (area under the receiver operating characteristic curve [AUROC], 0.6917; odds ratio [OR], 6.64; 95% CI, 1.98 to 25.23; *P* = .0009) (Figs 3B and 3C). The two *VAX2* methylation sites also showed a moderate ability to predict the EGFR-TKI response (Data Supplement).

We further performed stratification analysis by classifying patients as those with *EGFR*-activating mutations and those without (Figs 3D-F). Increased methylation of *HOXB9* (cg13643585) was observed in PD patients. The OR (6.63) between disease control and progression in the *EGFR* mutation group was significant (*P* = .018, Fig 3D). For the *EGFR* wild-type group, the OR (7.59) was comparable, but the *P* value was .09, likely because of the small sample size (Fig 3E). On the other hand, DNA methylation of *VAX2* (Data Supplement) exhibited no discriminatory power in the *EGFR* mutation group (OR, 2.29 and 2.27; *P* = .31 and .17 for cg07300178 and cg23885415, respectively; Data Supplement) but performed better in the wild-type group (OR, 11.07 and 6.06; *P* = .011 and .046, respectively; Data Supplement).

### Validation of *HOXB9* as a DNA Methylation Marker

We conducted pyrosequencing to quantify the DNA methylation level of *HOXB9* (cg13643585) in an independent cohort of 163 patients with *EGFR*-activating mutations who were receiving EGFR-TKI therapy. Tumor DNA was obtained before treatment. The data in Table 3 confirmed the pattern of increased *HOXB9* methylation in the EGFR-TKI–resistant (PD) group compared with the disease control group (CR, PR, or SD). The PD group was ranked first in each quartile, and the one-sided rank-sum test for between-group differences indicated a significant difference (*P* = .036) (Data Supplement). In addition, analysis of the AUROC showed that *HOXB9* methylation statistically significantly increased the predictive precision of EGFR-TKI resistance for the DCR (Table 3). Overall, the ratio of disease control to PD was approximately 5.5:1. For patients with lower *HOXB9* methylation levels (< 4.5), the ratio of disease control to PD increased to approximately 8:1. In contrast, for patients with higher *HOXB9* methylation levels (> 4.5), the ratio was greatly reduced—only approximately 2.5:1 (OR, 3.06; 95% CI, 1.13 to 8.19; *P* = .02). This result confirmed the benefit of using epigenomic markers complementing DNA markers to identify subpopulations of patients with higher-than-average susceptibility to intrinsic EGFR-TKI resistance (Fig 3G).

**TABLE 3.**
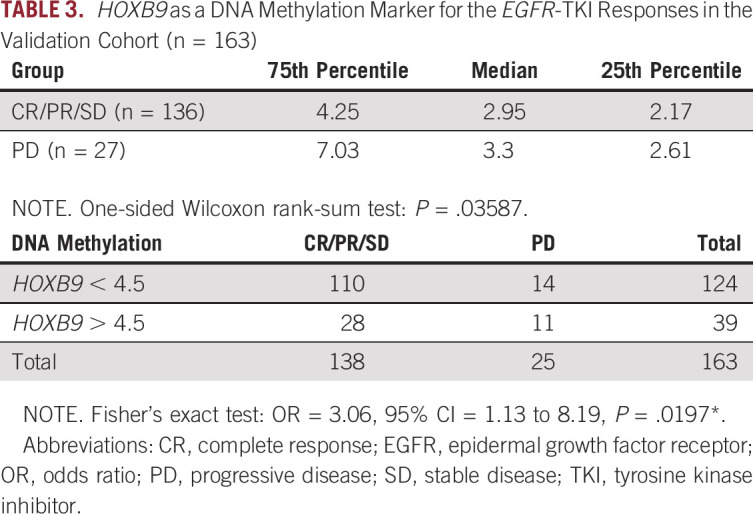
*HOXB9* as a DNA Methylation Marker for the *EGFR*-TKI Responses in the Validation Cohort (n = 163)

## DISCUSSION

The paradigm shift in treating lung adenocarcinoma with EGFR-targeted therapy is a major success in precision medicine. However, drug resistance remains a pertinent issue that hinders further improvement in the management of EGFR-targeted therapy. Although significant advances have been made in understanding acquired resistance (AR), the causes of IR remain unclear.^12^ In addition to being classified as AR versus IR, resistance mechanisms can be classified in terms of on-target versus off-target,^31^ suggesting the activation of collateral signaling. T790M mutation in exon 20 of *EGFR* is the most common mechanism for resistance to first- and second-generation EGFR-TKI. Several third-generation *EGFR*-mutant selective TKIs such as AZD9291 (osimertinib) have been approved for *EGFR* T790M–positive NSCLC treatment.^12^ As Oxnard^32^ summarized, the well-known EGFR T790M erlotinib-resistance mutation occurs both as the result of subclonal presence of T790M followed by outgrowth and as a de novo consequence of mutation in persister cells. Existence of any subclonal variation of methylation for either AR or IR posts an interesting issue to explore further. Moreover, where genomic resistance has been found, epigenomic modulation has been proposed as the potential mechanism. Changes in resistant phenotypes, including epithelial-mesenchymal transition (EMT) and cancer stemness shift, have been found to be driven by epigenetic remodeling. TKI-induced DNA methylation changes have been reported in advanced *EGFR*-mutated lung cancer.^33^ Decitabine, the DNA methyl transferase inhibitor, could reverse the sensitivity of EGFR-TKI–resistant NSCLC cell line PC9/GR through demethylation of *RASSF1A* and *GADD45β*.^34^ The combination of TKIs with epigenetic drugs has shown promise as a treatment in preclinical and clinical studies.^33-35^

In this study, we conducted clinical oncological investigation on the potential role of DNA methylation in mediating IR to EGFR-TKI treatment in patients with advanced lung adenocarcinoma. Aberrant DNA methylation is one of the most classical events that occurs during lung cancer development.^36^ Many studies have shown altered methylation patterns in lung cancer, indicating roles of epigenetic biomarkers and therapeutic targets.^37-39^ Earlier study by Zhu et al^40^ focused on the methylation patterns of Wnt antagonists, showing the association of methylated *SFRP5* with shortened progression-free survival under EGFR-TKI treatment, but not with IR to TKI. Epigenome-wide analysis has demonstrated that homeobox genes can act as potential DNA methylation markers for the early diagnosis of lung cancer.^41^ Moreover, Sandoval et al^42^ identified a hypermethylated five-gene signature associated with shorter relapse-free survival times of patients with stage I NSCLC without adjuvant chemotherapy. Interestingly, two of those five genes, *AXL1* and *HOXA9*, are homeobox TFs. Our pursuit of the primary EGFR-TKI–resistant methylation markers also identified enrichment of homeobox genes. Among the 30 TKI-associated methylation probes annotated to TFs, 11 accounted for nine homeobox genes (Data Supplement).

We identified and confirmed the correlation of *HOXB9* DNA methylation with an increased rate of IR to EGFR-TKIs. HOXB9 is involved in cell development and proliferation^43^ and is suggested to function as a TF that can induce the expression of EMT genes and several angiogenic factors, such as VEGF, IL-8, and TGFβ, resulting in the activation of *EGFR* and *ERBB2*.^44-46^ EGFR signaling is connected to the NF-κB pathway, giving the role in IR or AR to EGFR inhibitors.^47^ However, the molecular mechanisms by which HOXB9 contributes to carcinogenesis are debated.^48,49^ The overexpression of HOXB9 can suppress the AKT/NF-κB/Snail pathway and inhibit the proliferation of gastric carcinoma cells.^50^ We analyzed the correlation between EGFR signaling and NF-κB–dependent pathways (GSE60644) and found that expression of HOXB9 negatively associated with that of KIAA1199 (Cell migration–inducing hyaluronidase 1; Data Supplement). Through protein-protein interaction (PPI) analysis, we found that HOXB9 might cross talk with both IR and AR to EGFR-TKI through EZH2, SIRT1, and EGR2 (Data Supplement). Therefore, the regulation of HOXB9 is crucial in the cooperated oncogenic loops.^12^ Our data suggested that *HOXB9* hypermethylation may be a novel tumor cellular state that is useful for precise categorization of tumor heterogeneity in the study of intrinsic EGFR-TKI resistance via off-target effects such as redundant or compensating signaling. In addition, the pattern was consistent between patients with *EGFR*-activating mutations and patients with wild-type *EGFR*, implying that the regulatory effect of DNA methylation of *HOXB9* may be independent of *EGFR* activity.

DNA methylation changes can be accurately detected in tumors and liquid biopsies. Such detection is promising for the development of biomarkers for cancer screening.^51^ DNA methylation in distal regulatory sites, such as enhancer regions, plays important roles in gene regulation through the binding of cell type–specific TFs and interaction with promoters.^52-54^

We validated a DNA methylation site in the enhancer region of *HOXB9* that can help the prediction of nonresponse to EGFR-TKI. In cancer, aberrant DNA methylation at enhancers couples with recruitment of coactivators or corepressors, forming networks of cancer-associated TFs and their targeted genes.^55-57^ Stone et al^58^ defined hypermethylation enhancers that correlate with sensitivity to endocrine therapy, suggesting the impact of enhancer status on the drug treatment response. Therefore, DNA methylation at enhancers could regulate downstream gene expression, although the underlying mechanisms require further study.

The combination of genetic aberrations, gene expression, and DNA methylation highlights the potential of the identified candidates in the development of biomarkers for tumor diagnosis or prognosis. Additionally, clinical applications of biomarkers in public health, including the effect size, therapeutic drugs, or measurable signals, need to be considered. In this study, with a focus on medical actionability, we discovered that *HOXB9* methylation could be a biomarker useful for discriminating patients with TKI resistance from those with TKI sensitivity, especially patients whose tumors harbor *EGFR*-activating mutations. Although improving the sensitivity and specificity of *HOXB9* methylation is recommended, our work provided a preliminary proof of concept on the usefulness of *HOXB9* methylation for opening up more clinical options to manage lung adenocarcinoma. For example, in accordance with the current clinical standard of treating *EGFR*-mutant patients with EGFR-TKI, for patients with *HOXB9* hypermethylation, combination treatment such as EGFR-TKI plus antiangiogenic therapy^59^ or EGFR-TKI plus chemotherapy^60^ may be another option to overcome the resistance and improve the response rate. A larger cohort study with the inclusion of *HOXB9* methylation in addition to other genomic aberrations may be designed to investigate how to select patients for combination treatment.
